# US-guided percutaneous irrigation of extra-shoulder calcific tendinitis

**DOI:** 10.1093/bjr/tqad020

**Published:** 2023-12-12

**Authors:** Domenico Albano, Umberto Viglino, Carmelo Messina, Stefano Fusco, Salvatore Gitto, Francesca Lacelli, Luca Maria Sconfienza

**Affiliations:** IRCCS Istituto Ortopedico Galeazzi, Milan 20161, Italy; Dipartimento di Scienze Biomediche, Chirurgiche ed Odontoiatriche, Università degli Studi di Milano, Milan 20122, Italy; Dipartimento di Scienze della Salute, Scuola di Scienze Mediche e Farmaceutiche, Università di Genova, Genoa 16132, Italy; IRCCS Istituto Ortopedico Galeazzi, Milan 20161, Italy; Dipartimento di Scienze Biomediche per la Salute, Università degli Studi di Milano, Milan 20122, Italy; Dipartimento di Scienze Biomediche per la Salute, Università degli Studi di Milano, Milan 20122, Italy; IRCCS Istituto Ortopedico Galeazzi, Milan 20161, Italy; Dipartimento di Scienze Biomediche per la Salute, Università degli Studi di Milano, Milan 20122, Italy; ASL2 Dipartimento di Diagnostica - Radiologia P.O. Ponente, Pietra Ligure 17027, Italy; IRCCS Istituto Ortopedico Galeazzi, Milan 20161, Italy; Dipartimento di Scienze Biomediche per la Salute, Università degli Studi di Milano, Milan 20122, Italy

**Keywords:** calcific tendinopathy, ultrasound-guided percutaneous irrigation, tendon, gluteus, pain

## Abstract

**Objectives:**

To investigate the efficacy and safety of ultrasound-guided percutaneous irrigation of calcific tendinopathy (US-PICT) applied out of the shoulder, comparing its effectiveness to US-PICT of the rotator cuff.

**Methods:**

Patients subjected to US-PICT for extra-shoulder calcific tendinitis (Case Group) were compared to those subjected to US-PICT of the rotator cuff (Control Group). We had pre-procedure Visual Analogue Scale (VAS) pain score, 1- and 3-month VAS of patients of the Case Group, pre-procedure and 3-month VAS of patients of the Control Group.

**Results:**

The Case Group consisted of 41 patients (27 women; mean age: 45 ± 9years): 26 gluteus medius, 5 patellar tendon, 3 rectus femoris, 2 gluteus maximus, 2 common extensor tendon, 1 extensor carpi radialis longus, 1 pes anserinus, and 1 peroneus longus. The Control Group included 41 patients (27 women; mean age: 47 ± 11 years). The mean pre-procedure VAS of the Case Group was 8.8 ± 0.7 with a significant (*P* < .001) drop at 1 month (4.5 ± 0.6) and 3 months (3.6 ± 0.6). The mean pre-procedure VAS of the Control Group was 8 ± 1.4 and dropped to 3.1 ± 1.6 after 3 months (*P* < .001). Post-treatment VAS at 3 months was not significantly different between two Groups (*P* = 0.134). Similarly, the decrease of VAS from baseline to 3 months was not significantly different between the two Groups (*P* = 0.264).

**Conclusions:**

US-PICT is a safe and effective procedure that can be used out of the shoulder.

**Advances in knowledge:**

This study demonstrated the safety and effectiveness of US-PICT as a valuable therapeutic option for extra-shoulder calcific tendinitis, with similar clinical outcome to the same procedure performed in the rotator cuff. The technique must be adapted in some deeply located calcifications by means of the use of different needles and by thoroughly planning the access point for the procedure.

## Introduction

Calcific tendinitis is common and is related to the pathologic deposition of calcium hydroxyapatite crystals within the tendons. The most affected regions are the shoulder, mainly occurring in the supraspinatus tendon, and the hip, but different sites may be affected, and calcific tendinitis may occur near any tendinous insertion in the body.^1–3^ The process is unique and distinct from degenerative tendon changes. The cause is not completely understood but the pathogenesis is related to a hypoxic state of the tendinous fibres that leads to a reparative process with deposition of calcium hydroxyapatite within the tendon. Calcific tendinitis may be totally asymptomatic or cause mild pain, but, in the resorptive phase, it can be a non-negligible cause of intense pain, generally not responding to common oral painkillers or anti-inflammatory drugs, and often leading patients to seek for emergency medical consultation.

Ultrasound-guided percutaneous irrigation of calcific tendinopathy (US-PICT) is a well-established procedure that can be used as a first-choice treatment approach of shoulder calcific tendinitis.^4–6^ It has shown to be superior to subacromial steroid injections in terms of pain relief and functional recovery, even presenting less adverse effects than extracorporeal shock wave therapy.^7^ However, only small case series have been reported concerning its application beyond the shoulder.^8–11^ Further, the outcome of this procedure applied for treating calcific tendinitis outside the shoulder has not been compared to the standard procedure performed in the rotator cuff.

The aim of this study was to investigate the efficacy and safety of US-PICT applied out of the shoulder, comparing its effectiveness to that of the same procedure performed in the shoulder.

## Methods

Our Institutional Review Board approved this retrospective study and waived the need for informed consent (Protocol RETRORAD). After matching imaging, laboratory, and surgical data, our database was completely anonymized to delete any connections between data and patients’ identity according to the General Data Protection Regulation for Research Hospitals.

### Study design

Data were collected from our Institution (IRCCS Istituto Ortopedico Galeazzi, Milano, Italy). Patients were specifically sent by a pertinent orthopaedic surgeon for being subjected to US-PICT from 2016 to 2022. All patients experienced calcific tendinopathy, where pain was evidently associated with the calcification following a thorough clinical evaluation. Before treatment, the indication to treat calcific tendinitis was confirmed by means of US performed by a radiologist with 7-20 years of experience in this interventional procedure. As routinely done at our Institution, before the procedure, the radiologist conveyed thoroughly to the patient the technique, its potential effectiveness, and potential complications (including fainting, seizures, bursitis, infection) that were specified in a written consent form provided to the patients. All patients were clinically evaluated before the procedure to record the severity of pain according to the Visual Analogue Scale (VAS), which is a validated 11-point scale (ranging from 0 to 10) often used for measuring musculoskeletal pain.^12^^,^^13^ All patients were contacted by phone 1 and 3 months later to evaluate the response to treatment. Inclusion criteria were: (1) patients with intact and symptomatic calcification, with a clinical picture justified by calcific tendinitis according to a pertinent orthopaedic evaluation; (2) calcific tendinitis located out of the shoulder; (3) availability of clinical data in terms of VAS pain score at 1 and three months follow-up calls. We excluded patients with: (1) asymptomatic calcification; (2) pain not related to calcific tendinitis; (3) tendon tears; and (4) calcification that appeared smaller than 5 mm, migrated or fragmented. Also, we did not include patients already subjected to other treatments for calcific tendinitis. All data of US-PICT performed on patients with extra-shoulder calcific tendinitis (Case Group) were compared with data obtained retrospectively from previous US-PICT of rotator cuff calcific tendinitis performed on patients with similar age and sex at our institution (Control Group). Patients from Control Group were retrieved from our Institutional database and were used as controls in this study. From the Control Group, we had baseline VAS score before treatment and VAS score at 3 months. Any complications occurring during and after the procedures were reported according to the Society of Interventional Radiology classification.^14^*A priori* sample size was calculated using the G*Power software (Version 3.1.9.6, Dusseldorf University, Germany). Using *d* = 0.8, alpha = .05, and beta = .95, each group should be composed by a minimum of 35 subjects each.

### US-PICT technique

US-PICT procedure was the same in Case Group and Control Group. It was done according to the well-established procedure performed in the shoulder for rotator cuff calcific tendinitis^15^^,^^16^ by three different interventional radiologists with 7, 15, and 20 years of experience in this technique. First, after accurate skin disinfection, local anaesthetic (approximately 10 mL of 2% lidocaine chlorhydrate) was injected under US guidance into the skin/subcutaneous tissue and around the calcification. Then, one or more needles were inserted within the calcification under US monitoring.^17^ The length of the needle (3 cm 16-gauge or 9 cm 18-gauge) was chosen based on the depth of the calcification. The calcification was washed with a 10 mL syringe of warm saline NaCl 0.9% (heated to 42 °C [107 °F]),^18^ with alternative pushing and releasing of the syringe plunger, by creating a reflux mechanism that enabled to withdraw the saline together with the calcium fragments. Rarely, we used more needles just in case we were not able to “open” the whole calcification with one needle and we were unable to wash some components. The procedure was stopped when the flushed solution was free of visible calcium. Last, 1 mL of methylprednisolone acetate was injected around the treated tendon. Concerning post-operative management, we recommended keeping ice on the skin intermittently during the first 24 h to reduce pain, swelling and inflammation. Oral painkillers were also advised for five days (1000 mg of paracetamol two/three times per day). US-PICT of the shoulder was done as already reported in previous papers.^15^^,^^16^

### Statistical analysis

Variables were summarized as mean ± standard deviation. Differences among variables were evaluated by Fisher’s exact test for categorical variables, and by non-parametric Mann-Whitney *U*-test for continuous variables. We used the Friedman test to compare VAS pain scores before treatment, VAS at 1 month, and VAS at 3 months, followed by a Wilcoxon *post-hoc* test. A *P*-value lower than .05 was considered as statistically significant. Statistical analysis was performed using the SPSS software (v. 27, IBM, Armonk, NY).

## Results

According to our criteria, 41 patients were included in the Case Group (27 women, 14 men; mean age: 45 ± 9 years, range 28-56), with symptomatic calcific tendinitis within 41 tendons: 26 gluteus medius, 5 patellar tendon, 3 rectus femoris, 2 gluteus maximus, 2 common extensor tendon, 1 extensor carpi radialis longus, 1 pes anserinus, 1 peroneus longus. Forty-one patients subjected to US-PICT for RCCT (27 women, 14 men; mean age: 47 ± 11 years, range 21-74) were included in the Control Group. There were no significant differences of age (*P* = .327) and sex (*P* = .592) between the Case Group and Control Group. No complications were observed during and after the procedures, just mild increase of pain in five patients in the hours after the procedures with relief within the 24 h.

We used the 9 cm 18-gauge needles in 31/41 patients of the Case Group (76%) with deeply located calcifications within the gluteus medius, rectus femoris, and gluteus maximus tendons, while the 3 cm 16-gauge needles have been used in the remaining 10 patients (24%). The mean pre-procedure VAS score of the Case Group was 8.8 ± 0.7 and dropped to 4.5 ± 0.6 after 1 month and to 3.6 ± 0.6 after 3 months (*P* < .001). The mean pre-procedure VAS of the Control Group was 8 ± 1.4 and dropped to 3.1 ± 1.6 after 3 months (*P* < 0.001). A statistically significant difference was observed between the two groups regarding the pre-procedure VAS score (*P* = .003), but no significant differences were observed between the Case Group and the Control Group in terms of post-treatment VAS at 3 months (*P* = .134). Similarly, the analysis of the decrease of VAS pain score from baseline to 3 months’ evaluation showed no significant differences between the two Groups (*P* = .264).

**Figure 1.**
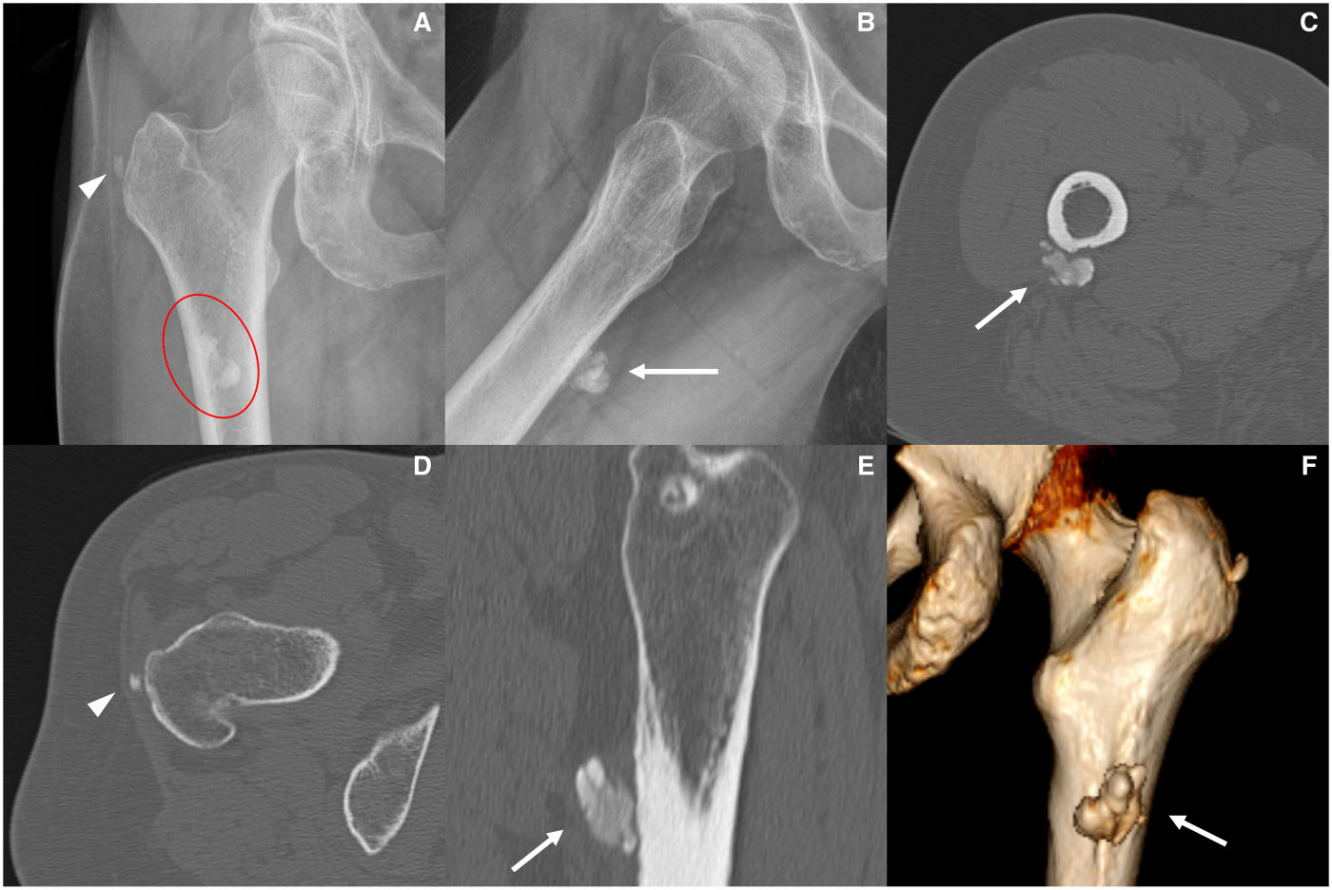
A case of calcific tendinitis of the trochanteric insertion of gluteus medius (arrowhead) and a bigger calcification at the femoral insertion of gluteus maximus (red circle and arrows) as seen in standard AP and Frog-leg radiographs (A, B) and in axial and sagittal CT images (C, D, E). The 3D reconstruction clearly depicts the localization of gluteus maximus calcification (F, arrow).

**Figure 2.**
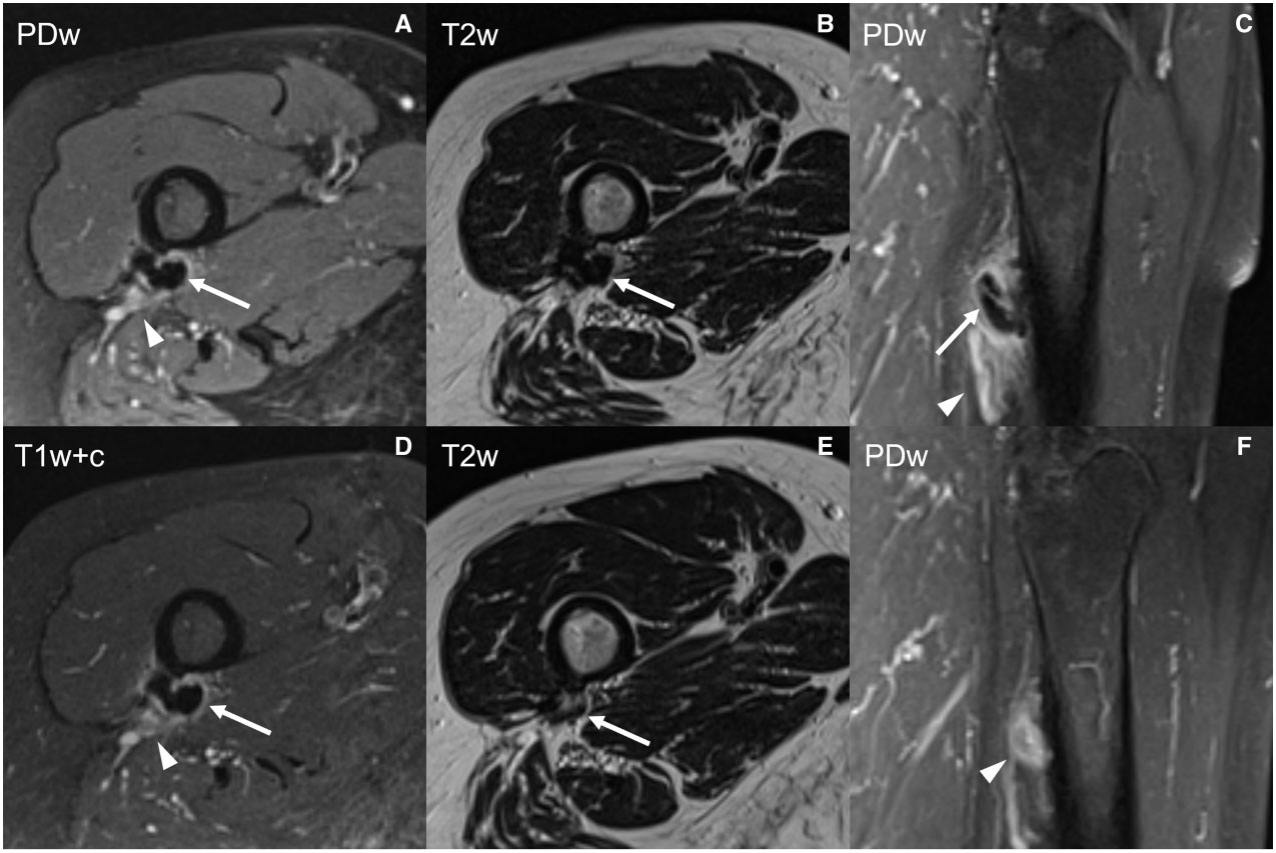
MRI of the same patient as shown in Figure 1; the calcification of gluteus maximus tendon (arrows) is hypointense in fat-suppressed proton-density (A, C), T2-weighted (B) and post-contrast fat-suppressed T1-weighted (D) images, with associated pericalcific oedema (A, C; arrowheads) and contrast enhancement (D; arrowhead). After US-PICT, the calcification is hardly recognizable (E; arrow) and scarce oedema is still observed (F; arrowhead). Abbreviations: PDw = fat-suppressed proton-density; T2w = T2-weighted; T1w+c = post-contrast fat-suppressed T1-weighted.

**Figure 3.**
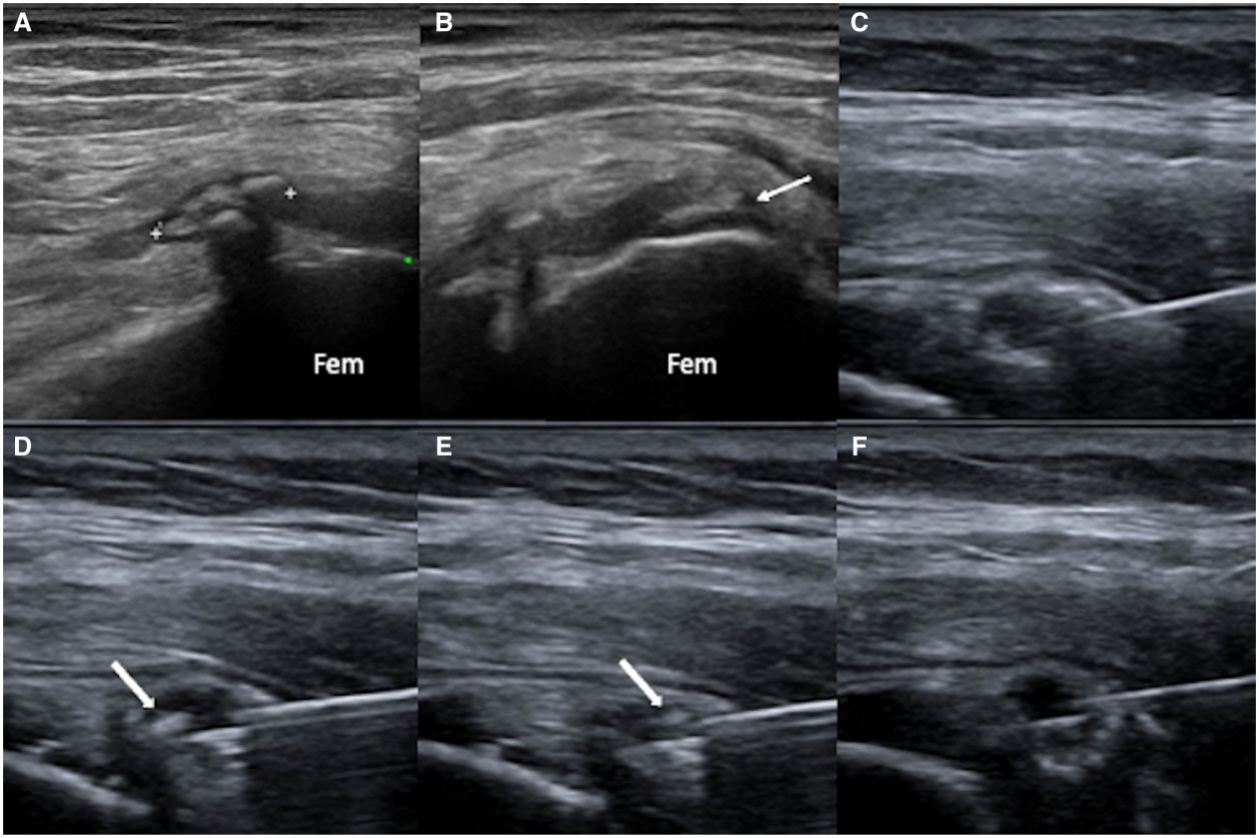
(A): a calcification of gluteus medius tendon at throchanteric insertion (*), with a smaller enthesopathic calcification (arrow) of the same tendon in (B). (C)-(F): US-PICT procedure of the bigger calcification, including needle insertion within the calcification (C), irrigation and washing (D, E) with a floating calcific debris (arrows). At the end of the procedure, there are no calcific deposits within the calcification (F).

**Figure 4.**
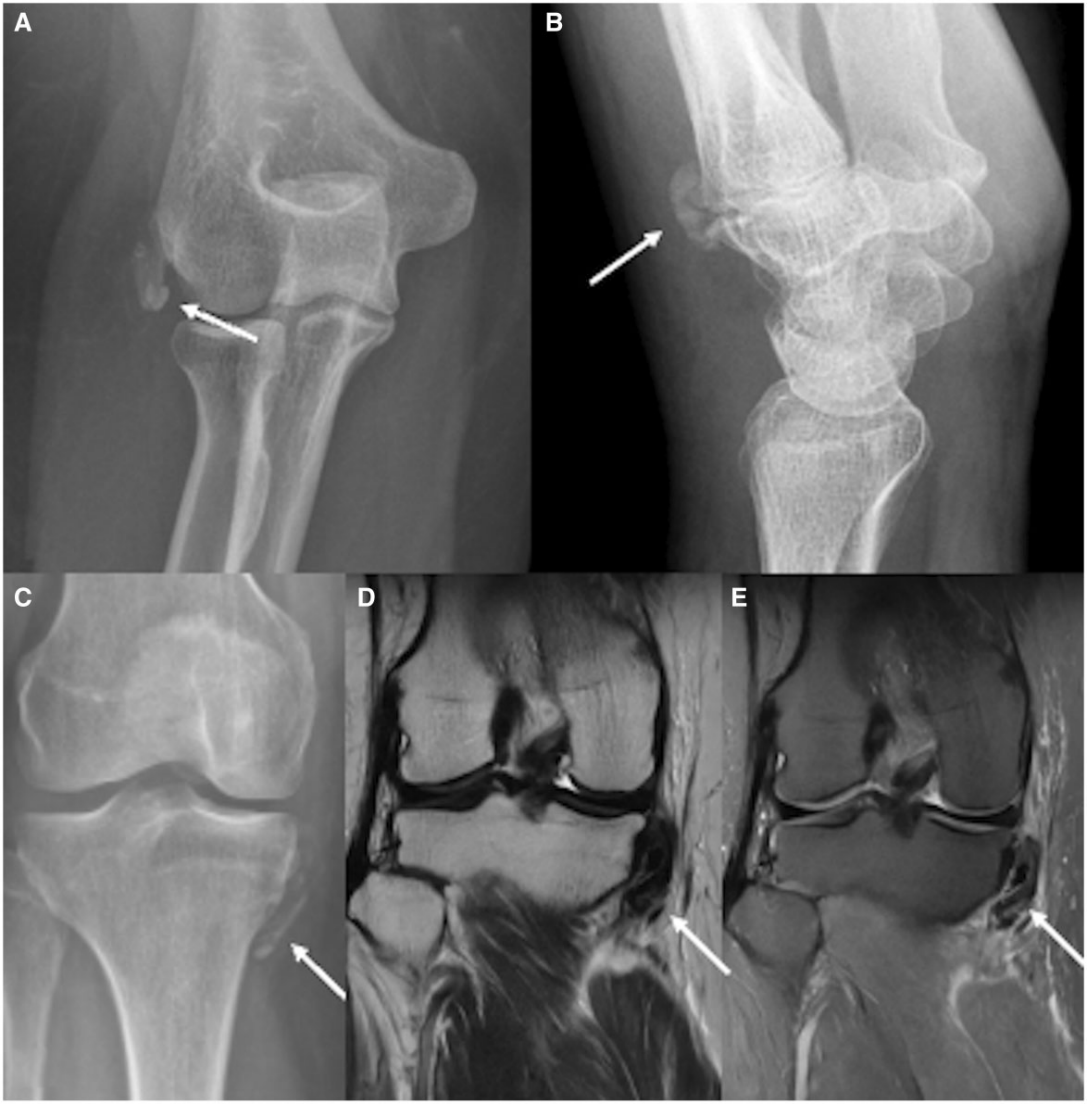
Some cases of calcific tendinitis outside the shoulder treated by US-PICT. Antero-posterior elbow radiograph (A) shows calcific tendinitis of common extensor tendon (arrow). Lateral view of wrist radiograph (B) shows calcific tendinitis of extensor carpi radialis longus tendon (arrow). Antero-posterior knee radiograph (C), coronal T2-weighted MR image (D) and fat-suppressed PD-weighted image (E) show calcific tendinitis of pes anserinus (arrows).

**Figure 5.**
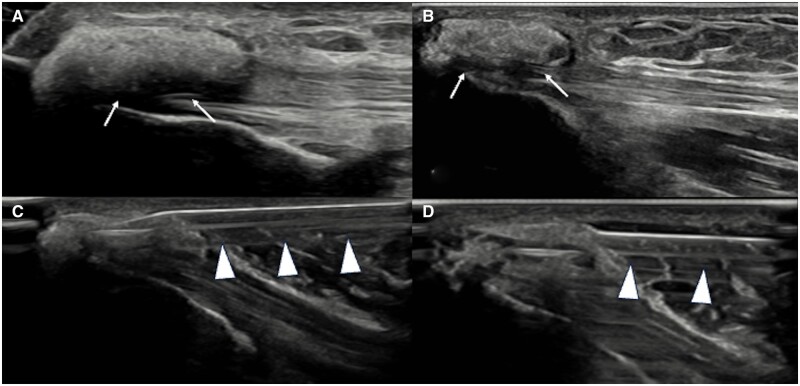
Ultrasound images (A, B) show a soft calcification (arrows) of triceps tendon. (C) and (D): US-PICT procedure including needle (arrowheads) insertion (C) within the calcification, irrigation and washing (D).

Figures 1- 5 show some representative cases of our study population.

## Discussion

Our main finding is the safety and effectiveness of US-PICT for extra-shoulder calcific tendinitis. Our 1-month and 3-month follow-up confirms the efficacy of the technique in short- and middle-term without any clinical complications. The decrease in VAS pain score the VAS pain at 3 months after US-PICT for both patients with shoulder and extra-shoulder calcific tendinitis were not significantly different.

Calcific tendinitis is a quite common cause of acute pain and in some cases can lead to access the emergency room but is often underdiagnosed. Ultrasound is usually not performed in acute setting for this kind of pain for the relatively low grade of urgency, and conventional radiographs are the usual diagnostic modality of choice. However, some of these calcifications can be undetectable because of small dimensions or due to projective reason. A proper ultrasound evaluation can discriminate the cause of the pain and might lead the patient to a US-PICT treatment when indicated.^4^^,^^7^^,^^19^

Calcific tendinitis develops in different stages^2^: from fibrocartilaginous transformation of a portion of the tendon in the precalcific stage to the proper calcific stage, which is composed of formative, resting, and resorptive phases. In the resorptive phase, the calcium deposits cause local inflammation with hyperaemia and migration of phagocytes that dissolute the calcific areas generating intratendinous oedema with intense acute pain. The last stage is the post-calcific stage where granulation tissue replaces calcium deposits.

Not every calcification is susceptible of treatment. Asymptomatic calcifications in resting stage do not usually require treatment. In case of mild symptoms without a significant reduction of quality of life, the first choice is conservative treatment with physical therapy and use of NSAIDs.^20^ In refractory cases, US evaluation is helpful for evaluating the eligibility to US-PICT.^21^ At the same time, calcifications that are fragmented or that have already been treated with other techniques, for example, extracorporeal shock wave therapy are usually not eligible of US-PICT treatment because the risk of calcific debris spreading in nearby tissues or bursae is high, causing more inflammation and pain.^22–25^ When indication and timing are correct, US-PICT is highly beneficial^16^^,^^21^^,^^26–29^ and our study demonstrates that US-PICT is a valid therapeutic option in extra-shoulder localizations where we obtained a reduction of VAS pain comparable to rotator cuff calcific tendinitis, in line with previous studies.^8–10^^,^^22^^,^^26^ The reduction or resolution of calcific tendinitis-related pain reduces the need of long-term anti-inflammatory or analgesic therapies.^26^^,^^30^ Notably, we have observed a progressive fragmentation and decrease in size and density of treated calcifications, as for the rotator cuff. Further, it can be necessary to perform the treatment again rarely, but it may occur just when large calcifications have not been washed completely. However, we have not encountered refractory cases in our practice, so US-PICT has always been performed once.

From a technical point of view, unusual locations imply the use of needles of different lengths and Gauge. Moreover, an accurate ultrasound planning is needed to have the best visualization of the needle and the best range of movement in cause when treating large calcifications. Gluteal and rectus femoris tendons need a 9 cm-long needle, instead of pes anserinus, patellar, peroneal, common extensor, and hand/wrist tendons that require the use of 3 cm-long needles. In some settings, higher gauges (19-23G) may be safer to reduce the risk of injury of neurovascular bundles in tricky areas (eg, wrist/hand), but we did not encounter any complications by even using 16-18G needles.

In case of calcific tendinitis, there is not a common consensus about the best therapeutic strategies after US-PICT. Several authors prefer to inject corticosteroid in the nearby bursa or peritendinous tissues, as we routinely do in shoulder and extra-shoulder calcific tendinitis. Others prefer to inject hyaluronic acid or platelet-rich plasma. Further, no consensus has been reached about post-treatment physiotherapy. Manual therapy and physiotherapy are valid conservative treatment options for calcific tendinitis of the shoulder, but it is not clear how they should be performed and for how long time.^31^^,^^32^ This is another interesting point that should be investigated for a better management of patients subjected to US-PICT outside the shoulder.

Extra-shoulder localizations are less common than rotator cuff calcific tendinitis,^1^^,^^2^ and in literature there are few scientific works about treatment.^8–11^ Compared to literature, our study has a larger group of patients and shows similar results to US-PICT procedures performed on patients with rotator cuff calcific tendinitis, with great results even in unusual locations in the hand and fingers. According to our data, the most common localization of extra-shoulder calcific tendinitis is the gluteus medius tendon, followed by patellar and rectus femoris tendon. This is not in line with the study by Spinnato et al,^9^ in which the most affected tendons were pectoralis major, proximal deltoid, elbow common extensor tendon, and posterior tibial tendon. This is probably due to the strongly active hip orthopaedic department of our institution that sends us several patients for interventional procedures.

One limitation of this study is the relatively small sample size with few cases in some localizations. This is probably because a lot of these conditions are underdiagnosed. Further, orthopaedists are scarcely aware about the opportunity of this safe and affective procedure outside the shoulder. Another limitation is the retrospective nature of the study, so prospective studies are warranted to compare the procedure with other conservative approaches and to understand how to manage these patients post-operatively.

In conclusion, US-PICT can be used for treating calcific tendinitis outside the shoulder. The technique must be adapted in some deeply located calcifications by means of the use of different needles and by thoroughly planning the access point for the procedure. Radiologists and other physicians should increase their knowledge about this condition, the possibility of extra-shoulder locations and the opportunity given by US-PICT as an effective and safe therapeutic option.
